# Body composition and neonatal nutrition research

**DOI:** 10.1038/s41390-026-04802-1

**Published:** 2026-02-05

**Authors:** Neena Modi

**Affiliations:** Imperial College London, London, UK

Christoph Binder and colleagues have carried out a secondary analysis of a cohort of infants born below 28 weeks gestation.^1^ The objective was to investigate the association between body composition assessed using whole body plethysmography at term-equivalent and growth between birth and term-equivalent, with neurodevelopmental outcomes assessed using the Bailey III at ages 1, 2, and 3 years.

The investigators found the Fat-Free-Mass (FFM) Z-score at term-equivalent was significantly and independently associated with higher Bayley-III scores across the domains of cognitive, language, and motor development throughout the first 3 years of life. In contrast, Fat-Mass (FM) Z-score and changes in weight, length, and head circumference Z-scores from birth until term were not significantly associated with neurodevelopmental outcomes. The authors conclude that inpatient gains in FFM appear to be critical for promoting favourable long-term neurodevelopment.

The authors are to be congratulated on conducting a meticulous longitudinal study in extremely preterm babies, a group at known high risk for adverse neurodevelopmental outcomes. What implications do their findings have for patient management today? Should, as the authors imply, nutritional care be directed towards increasing FFM?

The first and most important point, rightly highlighted by the authors, is that this is an observational study that has demonstrated associations. An association between variables should never be conflated with a causal relationship, as to do so risks drawing conclusions that may be invalid. It is entirely possible that the associations between FFM and more favourable neurodevelopment are driven by factors other than macronutrient intake. Plausible confounders include, for example, protein quality, intercurrent illness, and metabolic stability. It is therefore important to recognise that an association between FFM and better neurodevelopmental outcomes should not be taken to mean that nutritional strategies to increase FFM accretion will necessarily improve neurodevelopment. This hypothesis requires testing in well-designed randomised controlled trials.

The study findings are complex; for example, changes in length Z-Score showed statistically significant positive correlations with cognitive and motor domains at 1, 2, and 3 years of age but not with overall Bayley-III scores. However, a clear finding was that across all domains, changes in weight and head circumference Z-scores from birth until term-equivalent age showed no significant or independent association with Bayley-III scores through 3 years of age. That growth at term does not predict later neurodevelopment has been shown by other authors,^2^ so the finding by Binder et al provides important corroboration with clear implications for clinical management. Many neonatal unit nutritional regimens aim to promote third-trimester growth that is faster than intrauterine velocity in order to force very preterm babies to attain by term-equivalent, the size of babies born at full term, in the hope that this will promote better neurodevelopment. The conclusion from the study by Binder et al and others is that this logic is flawed. Infants born preterm generally weigh less than their counterparts that remain in utero, in keeping with the fact that preterm birth is indicative of difficulties with the pregnancy and intrauterine environment.^3^ Therefore, following birth, a preterm infant growing at intrauterine velocity will inevitably weigh less than a counterpart born at full-term.^4^ The study that is required to resolve the conundrum of optimal growth velocity for preterm infants is a randomised comparison of nutritional regimens that aim to achieve term size by term-equivalent age with those that support growth at intrauterine velocity.

In any study involving convenience samples and multiple testing, there is always a possibility that a statistically significant finding may have arisen by chance. The study was inevitably open to selection bias as infants who were not clinically stable or who were receiving respiratory support and therefore unable to undergo the body composition assessment were excluded, as were those with major periventricular haemorrhage. It is also not clear if the authors adjusted statistical probabilities for multiple testing. This notwithstanding, the study by Binder and colleagues illustrates that any randomised trial of the effect of nutritional strategies on neurodevelopment should be powered to detect pre-defined minimum clinically important differences in cognition, language, and motor development, ideally at age 5, when developmental attainments have stabilised.

It has been estimated that less than half of the variance in growth in preterm babies is attributable to nutritional intake,^5^ with the remainder the consequence of other influences, chief among which is intercurrent illness. This neonatal unit followed a well-defined nutritional guideline involving macronutrient fortification and the use of enriched formula to promote catch-up growth. The majority (69%) of infants received an exclusive human milk diet. Data are not provided on the extent to which this comprised the mother’s milk or pasteurised donor milk, nor the extent of use of fortifier manufactured from highly processed commercially sourced human milk. These are important considerations as fresh own mother’s milk contains a large number of non-nutritive growth-promoting factors, whereas pasteurisation and processing destroy or substantially reduce these components.^6^ It is relevant, therefore, that in the Binder study, the median Bayley Scales-III at 2 and 3 years of age, across cognitive, language, and motor domains, were below 85, indicating developmental impairment. The cardinal implication, also acknowledged by the authors, is that the nutritional strategies that promote optimum neurodevelopmental, cognitive, and metabolic outcomes are not known. The corollary is that current preterm nutritional guidelines, some of which are highly detailed and prescriptive, require robust evaluation and should be considered experimental, not standard of care.

The biological plausibility of the suggestion that preterm nutritional stratagems should be directed toward FFM deposition merits consideration. Substantial adipose tissue is laid down during the third trimester of human development and in early infancy, and human infants are among the most adipose of all mammalian species.^7^ It has been postulated that this phenomenon drove human evolution by providing a stable energy store to meet the demands of the rapidly growing infant brain.^7^ Further indication of the importance of human adiposity is that breastfeeding, in comparison to formula-feeding, appears to result in significantly higher FM and lower FFM up to 6 months of age.^8^ Breastfeeding also leads to better neurocognitive outcomes. Optimal body composition may therefore be reflected by the ratio of FM to FFM, rather than their absolute quantities.

Another area of importance and uncertainty is the emerging evidence that high protein intakes may be damaging to the preterm brain and to metabolic health. In a high-quality randomised controlled trial comparing an extra gram of amino acids administered parenterally in the first week after birth to babies below 1000 g birth weight (3.4 ± 0.6 g/kg/day compared with 2.6 ± 0.6 g/kg/day), language scores were lower in the higher intake group, and there were more children with moderate-to-severe neurodisability and cognitive delay.^9^ The same research group conducted a systematic review and meta-analysis of outcomes in relation to protein intakes above and below 3.5 g/kg/day. They found that infants receiving intakes above 3.5 g/kg/day had poorer neurodevelopmental and metabolic health outcomes.^10^ A high-quality randomised controlled trial involving term neonates showed that higher protein intakes result in a greater risk of childhood obesity.^11^ Other studies have highlighted the risks of amino acid intakes of no more than around 3.5 g/kg/day, especially in critically ill infants. These include lower morbidity-free survival, higher sepsis rates, and higher mortality^12–14^ The clinical implication of data such as these is that, though not definitive, caution in delivering higher amino-acid/protein intakes is warranted. Protein also requires adequate non-protein energy for satisfactory utilisation; optimal protein intakes are not known, and too much is as likely to be as harmful as too little.

In conclusion, Binder and colleagues have made a valuable contribution to the literature. Their investigation helps define the studies that are urgently needed to determine optimal nutritional strategies for extremely preterm babies and end an era of opinion-based practice. These studies should focus not on growth, body composition, or surrogate markers, but on neurodevelopment and other measures of functional health.
